# Validation of Artificial Intelligence Models for the Search, Selection of Articles, and Data Extraction in a Systematic Review and Meta-Analysis on Social Media and Video Games in Child and Adolescent Populations

**DOI:** 10.1192/j.eurpsy.2026.10963

**Published:** 2026-07-31

**Authors:** M. Martín De Argila Lorente, B. Rabinovici Gherman, L. Navarro Velasco, I. García Cabeza, F. Ferre Navarrete, D. Fraguas Herráez

**Affiliations:** 1 Hospital Dr. Rodríguez Lafora; 2 IBM; 3 Institute of Psychiatry and Mental Health, Hospital General Universitario Gregorio Marañón CIBERSAM, School of Medicine, Universidad Complutense de Madrid; 4 Institute of Psychiatry and Mental Health, Hospital Clínico San Carlos, IdISSC, CIBERSAM; School of Medicine, Universidad Complutense de Madrid, Madrid, Spain

## Abstract

**Introduction:**

Addiction to social media and video games in children and adolescents is associated with negative mental health outcomes, such as depression, anxiety, ADHD, social isolation, and suicidal risk.

**Objectives:**

This research project has two main aims. First, to develop an artificial intelligence (AI)-based tool, grounded in language models, to automate key stages of a systematic review and meta-analysis (search, article selection, and data extraction). Second, to conduct a systematic review and meta-analysis on the prevalence of use, the impact on mental health, and the effectiveness of interventions for social media and video game addiction in child and adolescent populations. The analysis will be carried out through two approaches: the conventional procedure and the AI-based tool, enabling methodological comparison and assessment of the potential of artificial intelligence in the synthesis of scientific evidence.

**Methods:**

Study Selection

**Traditional:** Two researchers will screen articles in three phases: (a) titles and abstracts according to inclusion criteria; (b) full-text review with assessment of mental health outcomes and sufficient data; (c) exclusion of overlapping studies.

**AI-based:** A large language model (LLM) will process data in Excel (phase 1) and full texts in Markdown format (phase 2), generating outputs in JSON. The model will detect overlapping studies for exclusion (phase 3).

**Data Extraction**

**Traditional:** Two researchers will extract data on publication characteristics, demographics, type of addiction, comorbidities, interventions, and outcomes.

**AI-based:** The LLM will analyze PDFs converted to Markdown, extracting the same variables into JSON format.

A three-level meta-analysis will be conducted following Cochrane guidelines, using literature from PubMed, Scopus, MEDLINE, and the Cochrane Library, with direct comparison between both methods.

**Results:**

The AI-based tool is expected to automate the processes of search, selection, and data extraction for systematic reviews, reducing the required time and improving methodological efficiency. Furthermore, the systematic review and meta-analysis are anticipated to reveal significant associations between problematic use of social media and video games and various mental health indicators in children and adolescents, such as symptoms of anxiety, depression, ADHD, and suicidal behaviors. The comparison between the conventional approach and the AI-assisted approach will allow for the evaluation of the potential of these technologies in the synthesis of scientific evidence and their future applicability in clinical and academic settings.

**Conclusions:**

This study is pioneering in applying generative AI to systematic reviews. It also addresses an urgent child and adolescent mental health issue related to behavioral addictions.

**Disclosure of Interest:**

None Declared

